# An Identity-Based (IDB) Broadcast Encryption Scheme with Personalized Messages (BEPM)

**DOI:** 10.1371/journal.pone.0143975

**Published:** 2015-12-02

**Authors:** Ke Xu, Yongjian Liao, Li Qiao, Zhangyun Liu, Xiaowei Yang

**Affiliations:** School of Computer Science and Engineering, University of Electronic Science and Technology of China, ChengDu, SiChuan, China; Beijing University of Posts and Telecommunications, CHINA

## Abstract

A broadcast encryption scheme with personalized messages (BEPM) is a scheme in which a broadcaster transmits not only encrypted broadcast messages to a subset of recipients but also encrypted personalized messages to each user individually. Several broadcast encryption (BE) schemes allow a broadcaster encrypts a message for a subset *S* of recipients with public keys and any user in *S* can decrypt the message with his/her private key. However, these BE schemes can not provide an efficient way to transmit encrypted personalized messages to each user individually. In this paper, we propose a broadcast encryption scheme with a transmission of personalized messages. Besides, the scheme is based on multilinear maps ensure constant ciphertext size and private key size of each user and the scheme can achieve statically security. More realistically, the scheme can be applied to the Conditional Access System (CAS) of pay television (pay-TV) efficiently and safely.

## Introduction

The concept of broadcast encryption (BE) was first formally defined by Fiat and Naor in 1994 [1], which is a communication mode of public-key encryption to the multi-recipient. In BE schemes, a broadcaster encrypts broadcast messages and transmits them to a set *S* of users who are listening on a broadcast channel. Each user in set S uses his/her private key to decrypt the broadcast messages at the same time. Broadcast encryption has wide applications such as digital rights management, pay TV, satellite radio communication, video conference and wireless sensor network [2].

In general broadcast encryption schemes, a broadcaster first chooses a set *S* of users who will be able to decrypt broadcast messages as authorized users’ set and encrypts a computed secret broadcast key *K* into header as a part of ciphertext. Then it uses the secret key *K* to encrypt broadcast messages in a symmetric encryption way as the other part of ciphertext. Any user who is listening on a broadcast channel can receive the ciphertext with two parts. But only the user in set *S* can use his/her private key to decrypt the ciphertext to get the broadcast messages. A broadcast encryption scheme is said to be fully collusion resistant [3] when even if all users that are not in *S* collude, they can by no means infer any information about the broadcast message. For solving the certificate management, Shamir first presented the concept of the identity-based cryptosystems in [4]. An identity-based encryption (IBE) scheme enables users to set public keys related to their own identities like e-mails, telephone numbers and other arbitrary strings. Besides, IBE reduces initialization, computational overhead and intercommunication, simplifies key management and eliminates the need for private key database.

The pay television (pay-TV) broadcasting contains a Conditional Access System (CAS) where a broadcaster encrypts two kinds of messages to each user: Entitlement Control Messages (ECM) and Entitlement Management Messages (EMM). ECM is common information to all users and the transmission of ECM is similar to a general broadcast encryption way by using users’ public keys. EMM includes contract information for a particular user and each user’s private key is used to encrypt EMM in a symmetric encryption way. So the broadcaster must manage all of the users’ public keys as well as private keys. Hence, the key management cost of the broadcaster is larger than the general broadcast encryption schemes due to extra management of all users’ private keys. It is necessary to reduce the management cost of the broadcaster in one aspect: low overhead and efficient transmission of ECM and EMM. In [5] Aggelos pointed out the efficiency of a broadcast encryption scheme is according to four parameters: key-storage, decryption overhead, encryption overhead and transmission overhead. The ciphertext overhead of a broadcast scheme is defined in [6]: the number of bits in the ciphertext beyond what is needed for the description of the recipient set and the symmetric encryption of the plaintext payload. This shows a BEPM scheme is more efficient if the ciphertext overhead is shorter and the private key management cost is less and it will have low overhead if the ciphertext overhead depends at most logarithmically on the number of broadcast users.

Several broadcast encryption schemes [6–9] have been proposed and they all provide the transmission of broadcast messages like ECM. Especially in 2005, Boneh, Gentry, Waters [7] introduced an identity-based broadcast encryption scheme BGW which was against collusion resistance and the length of ciphertext and private key were constant. In 2012, Yanli Ren [10] constructed a dynamic identity-based broadcast encryption scheme which had a tight security reduction without random oracle. In 2003, the multilinear maps were firstly defined by Silverberg and Boneh in [11], and they showed three properties about multilinear maps which were useful to construct multiparty key exchange and broadcast encryption schemes. In [6], Boneh et al used multilinear maps to construct three low overhead BE schemes with shorter public key size than any previous BE schemes. And in [12], Boneh first used indistinguishability Obfuscation (iO) to construct a distribute BE scheme in which the ciphertext size was independent of the number of recipients. These schemes above can provide secure communication between a broadcaster and a group of users and the broadcaster encrypts content like ECM by simply using public keys of the broadcaster and recipients. The key management cost of these schemes is very small because of the openness of public keys. However, these schemes cannot be used by the broadcaster to transmit personalized messages which are different like EMM to individual users at the same time.

In 2002, Kurosawa [13] defined a multi-recipient encryption scheme as a particular public key encryption scheme which can provide transmission of personalized messages to each user efficiently. In 2009, Harunaga [14] constructed a multi-recipient public key encryption scheme to send personalized messages to each user individually. However, it is inefficient for a sender to transmit the broadcast messages (identical personalized messages) to each user respectively on these schemes. Until now, there was only one scheme constructed by Ohtake [15] that can achieve the function of a broadcaster can encrypt not only broadcast messages but also personalized messages for recipients. But its public key size is the number of 3*n* + 2 elements of group $G_{1}$ ($G_{1}$ is a group of prime order *p* and *n* is the total number of recipients) and it is based on Public Key Infrastructure (PKI) rather than identity-based. Hence, our goal here is to construct a low overhead and identity-based BEPM which can be used in CAS efficiently by using multilinear maps.

### Our Contributions

In this paper, we describe an identity-based BEPM scheme that uses asymmetric multilinear maps constructed by Boneh in [6] and extends their BE scheme. Our scheme reduces the ciphertext length in general muiti-recipient encryption schemes and the public key size in other BEPM schemes. Compared with the existed scheme, our scheme reduces the management cost of public keys and private keys. In addition, the public key size in our scheme is shorter than the other existed schemes [6, 15] and each user’s private key and ciphertext are still in constant size. Besides, we prove that our scheme is statically-secure under the decisional n-Hybrid Diffie-Hellman Exponent problem (*n*-HDHE) and that it is efficient to be applied to CAS. Our scheme is fully collusion-resistant against any number of colluders.

### Organization

The rest of our paper is organized as follows: we will recall some related definitions in section 2. We show the detailed construction of our identity-based BEPM scheme in section 3. We will analyze the security of our scheme and give the comparison between our scheme and the other schemes in section 4. Finally, we will apply our scheme to CAS in section 5.

## Preliminaries

### Asymmetric Multilinear Maps

We use the asymmetric multilinear maps constructed in [6]. It uses $g_{i}^{a}$ to represent a levle-*i* encoding of *a*. then the map *e* can combine a level *i* encoding and a levle *j* encoding to generate a level *i* + *j* encoding. It uses integer vectors rather than integers to index groups. The detailed algorithms are as follows:

**Setup** ($\vec{n}$). Use a some positive integer vector $\vec{n}$ and set up a $\vec{n}$-linear map. Let *p* be a large prime number, it outputs a description of groups $G_{\vec{v}}$ of prime order *p* and $\vec{v}$ are non-negative integer vectors where $\vec{v}\leq \vec{n}$. It also outputs a description of generators $g_{\vec{v}}\in G_{\vec{v}}$. In addition, set $G_{{\vec{e}}_{i}}$ be the *i*th source group and ${\vec{e}}_{i}$ be a standard basis vector in the group $G_{{\vec{e}}_{i}}$ which means ${\vec{e}}_{i}=(0,...,1,...,0)$ is a vector of *n* 0*s* and 1 in the *i*th place. $G_{\vec{n}}$ is the target group and the rest of the $G_{\vec{v}}$ groups are intermediate groups. So it can get the following map operations:

Input two elements $h\in G_{{\vec{v}}_{2}}$ and $g\in G_{{\vec{v}}_{1}}$ with ${\vec{v}}_{1}+{\vec{v}}_{2}\leq \vec{n}$, it outputs an element of $G_{{\vec{v}}_{1}+{\vec{v}}_{2}}$. It can get the map operation $e_{{\vec{v}}_{1},{\vec{v}}_{2}}(g,h):e_{{\vec{v}}_{1},{\vec{v}}_{2}}(g_{{\vec{v}}_{1}}^{a},g_{{\vec{v}}_{2}}^{b})=g_{{\vec{v}}_{1}+{\vec{v}}_{2}}^{ab}$. It omits the subscript and write *e*_2_ to represent the pairing operation of two element in group. It generalizes *e*_2_ to multiple inputs as *e*(*h* ^(1)^, *h* ^(2)^, …, *h* ^(*k*)^) = *e*(*h* ^(1)^, *e*(*h* ^(2)^, …, *h* ^(*k*)^)). So it writes *e*_*n*_ to represent the multiple operation of *n* elements.

The asymmetric multilinear maps can satisfy three properties introduced in [11]: multilinearity, non-degeneracy and computability.

### Hardness Assumption

We recall the definition of decisional *n*-Hybrid Diffie-Hellman Exponent. The detailed definition is as follows:

Let $\vec{n}$ is the all-ones vector of *n* + 1 length, $\vec{e}$ is a *n* + 1 length vector of *n* 0*s* and 1 in the *i*th place and the multilinear map *e* has the source group $G_{\vec{n}}$ and target group $G_{2\vec{n}}$. We randomly choose $\alpha \in {\mathbb{Z}}_{p}$ where *p* is a large prime number. Let $X_{i}=g_{{\vec{e}}_{i}}^{\alpha^{2^{i}}}(i=0,\ldots ,n-1)$ and $X_{n}=g_{{\vec{e}}_{n}}^{\alpha^{2^{n}+1}}$. Then choose a random $t\in {\mathbb{Z}}_{p}$ and let $V=g_{\vec{n}}^{t}$. We now define the decisional *n*-Hybrid Diffie-Hellman Exponent assumption as given {*X*_*i*_}(*i* = 0, …, *n* − 1), *V* and the $K=g_{2\vec{n}}^{t\alpha^{n}}$ or *K* = K* as K* is a random element in group $G_{2\vec{n}}$.

**Definition 1** *We say the decisional n-Hybrid Diffie-Hellman Exponent assumption is hard as any polynomial n and probabilistic polynomial time (PPT) algorithm* A *has negligible advantage to distinguish* $K=g_{2\vec{n}}^{t\alpha^{n}}$ and K = K*.

### broadcast encryption with personalized message

We first introduce the definition and the security model of the identity-based BEPM. An identity-based BEPM scheme includes the following four algorithms:

**Setup** (ID). Set up an identity space ID for a BEPM scheme. It outputs public parameters *params* and master secret key *msk*.

**Extract**(*msk*, *u*). Take the master secret key *msk* and a user u∈ID, and it outputs a private key *sk*_*u*_ for user *i* with identity *u*.

**Enc**(*params*, *S*). Input the public parameters *params* and polynomial sized set S⊆ID of authorized recipients, and then produce a pair (*Hdr, K*) and a list of personalized keys as *K*_*u*_ for the user u∈ID. Use *Hdr* to guarantee the confidentiality of *K* which is the symmetrical encryption key used to encrypt broadcast messages as *c* and also *K*_*u*_ is a personalized symmetrical encryption key of user *i* with identity *u* used to encrypt a personalized message as *c*_*u*_. It finally outputs (*Hdr, c, c*_*u*_ *(u* *∈* *S)*) as a ciphertext.

**Dec**(*params*, *u*, *sku*, *Hdr*, *S*). The decryption algorithm inputs *Hdr* and the private key *sk*_*u*_ of user *i* with identity u∈ID, and outputs the key pair (*K, K*_*u*_) for user *i* with identity u∈ID. If u∉S, the decryption algorithm outputs ⊥. Otherwise, the user *i* decrypts the *Hdr* by using its private key *sk*_*u*_ to get *K* and *K*_*u*_, and finally decrypts the ciphertext *c* and *c*_*u*_ respectively.

For security, there are mainly two notions of security: statically secure under a chosen plaintext attack (CPA) and adaptively secure under an adaptively chosen ciphertext attack (CCA2). We define the CPA security as follows:

**Setup**. The challenger C runs Setup(ID) to get (*params*, *msk*) and gives *params* to A.

**Private Key Queries**. A adaptively makes private key queries for user *i* with identity u∈ID. The challenger C runs *Extract*(*params*, *msk*) to get private key *sk*_*u*_ and gives *sk*_*u*_ to A.

**Challenge**. A submits a set $S^{*}\subset ID$ and *u* ∉ S* for any *u* requested in a private key query. The challenger gets $\left(Hdr^{*},K_{0}^{*},{\left\{K_{u}^{*}\right\}}_{u\in S^{*}}\right)$ from *Enc*(*params*, S*). And if *b* = 0, the challenger gives $\left(Hdr^{*},K_{0}^{*}\right)$ to A, if *b* = 1, the challenger chooses a random key $K_{1}^{*}$ to A. Also, the challenger selects {*b*_*u*_}_*u* ∈ S*_, and if *b*_*u*_ = 0, it gives $K_{u}^{*}$ to A, if *b*_*u*_ = 1, the challenger also chooses a random key $K_{u}^{{*}^{\prime }}$ to A.

**More Private Key Queries** A adaptively makes private key queries for user *i* with identity *u* ∉ S*. The challenger C runs *Extract*(*params*, *msk*) to get *sk*_*u*_ and gives *sk*_*u*_ to A.

**Guess**. A makes a guess *b*′ for the random value *b*. And A also gives a list of guess ${\left\{b_{u}^{\prime }\right\}}_{u\in S^{*}}$ for {*b*_*u*_}_*u* ∈ S*_. |S*| is the total number of elements in set S*.

So we can get that the advantage of A is:

$$\begin{array}{c} Adv=|Pr\left[\left(b^{{}^{\prime }}=b\right)⋀{\left(b_{u}^{{}^{\prime }}=b_{u}\right)}_{\forall u\in S^{*}}\right]-\frac{1}{2}\times \frac{1}{2^{|S^{*}|}}| \end{array}$$

In CAS, a security module (smart card) is inserted into each user’s terminal and given to each user respectively. As the personalized message can only be decrypted in a security module, so no one can get a personalized message as a plaintext in CAS. Hence, we concentrate on the notion of CPA security as we shows below.

**Definition 2** *A BEPM scheme is said to be statically secure under a chosen plaintext attack if for any polynomial time adversary* A *that can not make any decryption queries and must determine the challenge set S before Setup, the advantage Adv is negligible*.

## Our Construction

In this section, we give our construction for an identity-based BEPM scheme based on multilinear maps in details.

First let *N* = 2^*n*^ − 1 (*n* is an integer) and $\vec{n}=(1,\ldots ,1)$ is a vector of *n* + 1 1s. Use an asymmetric multilinear map where $G_{\vec{n}}$ is the source group and $G_{\vec{2n}}$ is the target group of prime order *p* which means any two elements in $G_{\vec{n}}$ use *e*_2_ to map an element in $G_{2\vec{n}}$. From what set above, the asymmetric multilinear maps have the following properties:

1. For all standard basis vectors ${\vec{e}}_{i}\in G_{{\vec{e}}_{i}}$, we have a map *e*_*n*+1_ to group $G_{\vec{n}}:e_{n+1}(g_{{\vec{e}}_{0}},...,g_{{\vec{e}}_{n}})=g_{\vec{n}}$.
2. For any two elements $g_{\vec{n}}^{a}$ and $g_{\vec{n}}^{b}$ (*a*, *b* are integers) in group $G_{\vec{n}}$, we have a map *e*_2_ to group $G_{\vec{2n}}:e_{2}(g_{\vec{n}}^{a},g_{\vec{n}}^{b})=g_{\vec{2n}}^{ab}$.

**Setup**(*n*). *n* is the length of users’ identities. The identity space is $ID={\left\{0,1\right\}}^{n}$ except {0}^*n*^. It uses the multilinear map *e* constructed in section 2.1 to get vector $\vec{n}$, ${\vec{e}}_{i}(i=0,\ldots ,n)$, group $G_{\vec{n}}$ and group $G_{2\vec{n}}$. Then it randomly chooses $\alpha ,\{\beta_{i}\}\left(i=1,...,n\right),\gamma \in {\mathbb{Z}}_{p}$ and computes:

$$X_{i}=g_{{\vec{e}}_{i}}^{\alpha^{2^{i}}}(i=0,\ldots ,n-1),X_{n}=g_{{\vec{e}}_{n}}^{\alpha^{\left(2^{n}+1\right)}}$$

$$Y_{i}=g_{\vec{n}}^{\beta_{i}}(i=1,\ldots ,n),W=g_{2\vec{n}}^{\alpha^{2^{n}}},V=g_{\gamma }^{\vec{n}}$$

So it can get the public parameters and a master key as follows:

$params=(g,e_{2},e_{n+1},\{{\vec{e}}_{i}\}\left(i=0,...,n\right),p,G_{\vec{n}},G_{2\vec{n}},\vec{n},W,V,\{X_{i}\}\left(i=0,...,n\right),\{Y_{i}\}\left(i=1,...,n\right))$

*msk* = (*α*, *γ*, {*β*_*i*_}(*i* = 1, …, *n*))

**Extract**(*params*, *msk*, *u*). All users use their identities such as u∈ID as their public keys. And then the public key generator (PKG) gives the private key ${sk}_{u}=\left(sk_{u1},sk_{u2})=(g_{\vec{n}}^{\gamma \alpha^{u}},V^{\beta_{i}}\right)$ to the user *i* with the identity u∈ID.

**Enc** (*params*). Set an authorized set *S* of recipients. Then randomly choose $t\in {\mathbb{Z}}_{p}$, and for any u∈ID, and let *u*_*i*_ represents the *i*th position in the binary of *u*. Finally it computes as follows:

$$\begin{array}{c} Z_{u}=e_{n+1}\left(X_{0}^{u_{0}}g_{{\vec{e}}_{0}}^{1-u_{0}},\ldots ,X_{n-1}^{u_{n-1}}g_{{\vec{e}}_{n-1}}^{1-u_{n-1}},g_{{\vec{e}}_{n}}\right)=g_{\vec{n}}^{\alpha^{u}} \end{array}$$

where *u*_*i*_ ∈ {0, 1} and $X_{i}^{0}g_{{\vec{e}}_{i}}^{1}=g_{{\vec{e}}_{i}}$ and $X_{i}^{1}g_{{\vec{e}}_{i}}^{0}=X_{i}$.

$$\begin{array}{c} K=W^{t}, \end{array}$$

$$\begin{array}{c} Hdr=\left(h_{0},h_{1}\right)=\left(g_{\vec{n}}^{t},{\left(V\cdot \prod_{u\in S}Z_{2^{n}-u}\right)}^{t}\right), \end{array}$$

$$\begin{array}{c} K_{u}=e_{2}{\left(g_{\vec{n}}^{\beta_{i}},V\right)}^{t} \end{array}$$

for the user *i* with identity *u* in set *S*.

It finally outputs $\left(K,Hdr,{\left\{K_{u}\right\}}_{u\in ID}\right)$.

**Dec**(*params*, *S*, *Hdr*). The user *i* with identity *u* decrypts as follows: if *u* ∉ *S*, then output ⊥. Otherwise it lets *Hdr* = (*h*_0_, *h*_1_) and the receiver *i* with identity *u* and private key *sk*_*u*_ can compute *K* = *e*_2_(*Z*_*u*_, *h*_1_)/*e*_2_((*sk*_*u*_ ⋅ ∏_*j* ∈ *S*, *j* ≠ *u*_ *Z*_2^*n*^ − *j* + *u*_), *h*_0_) and the personalized key for user *u* is *K*_*u*_ = *e*_2_(*h*_0_, *sk*_*u*2_).

It now verifies the correctness of our scheme as follows:

$$\begin{array}{c} K=e_{2}\left(Z_{u},h_{1}\right)/e_{2}\left(\left(sk_{u}\cdot \prod_{j\in S,j\neq u}Z_{2^{n}-j+u}\right),h_{0}\right) \end{array}$$

$$\begin{array}{c} =e_{2}\left(g_{n}^{\alpha^{u}},g^{t(\gamma +\sum u\in S\alpha^{2^{n}-u})}\right)/e_{2}\left(g_{\vec{n}}^{\gamma \alpha^{u}}\cdot g^{\sum_{j\in S,j\neq u}\alpha_{\vec{n}}^{2^{n}-j+u}},g_{\vec{n}}^{t}\right)=g_{2\vec{n}}^{t\alpha^{2^{n}}}, \end{array}$$

$$\begin{array}{c} K_{u}=e_{2}\left(h_{0},sk_{u2}\right)=e_{2}\left(g_{\vec{n}}^{t},g_{\vec{n}}^{\gamma \beta_{i}}\right)=e_{2}\left(g_{\vec{n}}^{\beta_{i}},g_{\vec{n}}^{t\gamma }\right)=e_{2}\left(g_{\vec{n}}^{\beta_{i}},V^{t}\right). \end{array}$$

## Security Analysis

In this section, we prove the security of our BEPM scheme and show the comparison between our scheme and the other schemes.

### security

First, we show the security proof of our BEPM scheme as follows:

**Theorem 1** *Construct an asymmetric multilinear map e_n + 1_ and a map e_2_ for a vector* $\vec{n}$, ${\vec{e}}_{i}(i=0,...,n)$, *group* $G_{\vec{n}}$ *and group* $G_{2\vec{n}}$, *and assume the decisional n-Hybrid Diffie-Hellman Exponent assumption is hard for the multilinear map e_n + 1_. Then we can get that our identity-based BEPM scheme is statically secure*.

*Proof*. Assume that there is an adversary A who has advantage *ϵ* to break the BEPM scheme, and then we build an algorithm ℬ to solve the decisional *n*-Hybrid Diffie-Hellman Exponent problem. A and ℬ interact as follows:

**Setup**. ℬ constructs a multilinear map *e* as section 2.1 shows for vector $\vec{n}$, ${\vec{e}}_{i}(i=0,...,n)$, group $G_{\vec{n}}$, and group $G_{2\vec{n}}$ and chooses a random $\alpha \in {\mathbb{Z}}_{p}$ and a random $t\in {\mathbb{Z}}_{p}$ and then computes the public parameters as follows:

$$\begin{array}{c} X_{i}=g_{{\vec{e}}_{i}}^{\alpha^{2^{i}}}\left(i=0,\ldots ,n-1\right), \end{array}$$

$$\begin{array}{c} X_{n}=g_{{\vec{e}}_{n}}^{\alpha^{2^{n}+1}},U=g_{{\vec{n}}^{t}}, \end{array}$$

$$\begin{array}{c} W=e_{2}\left(e_{n+1}\left(g_{{\vec{e}}_{0}},...,g_{{\vec{e}}_{n}-2},X_{n-1},g_{{\vec{e}}_{n}}\right),e_{n+1}\left(g_{{\vec{e}}_{0}},...,g_{{\vec{e}}_{n}-2},X_{n-1},g_{{\vec{e}}_{n}}\right)\right). \end{array}$$

The adversary A submits the challenge users’ identities set *S* which is a subset of ID. And ℬ randomly chooses $r\in {\mathbb{Z}}_{p}$ to compute:

$$\begin{array}{c} V=g_{\vec{n}}^{r}/\prod_{u\in S}Z_{2^{n}-u}, \end{array}$$

$$\begin{array}{c} Z_{u}=e_{n+1}\left(X_{0}^{u_{0}}g_{{\vec{e}}_{0}}^{1-u_{0}},...,X_{n-1}^{u_{n-1}}g_{{\vec{e}}_{n-1}}^{1-u_{n-1}},g_{{\vec{e}}_{n}}\right)=g_{\vec{n}}^{\alpha^{u}}. \end{array}$$

Hence, *γ* = *r* − ∑_*u* ∈ *S*_ *α* ^2^*n*^ − *u*^ and *γ* is also uniform.

Finally ℬ gives the adversary A (*V*, *W*, {*X*_*i*_}(*i* = 0, …, *n*)).

**Private Key Queries**. The adversary A makes private key queries for users’ identities *u* ∉ *S*. Then ℬ responses as follows:

ℬ first randomly chooses $\beta_{1}^{\prime },..,\beta_{n}^{\prime },\theta_{1},...,\theta_{n}\in {\mathbb{Z}}_{p}$ and computes:

$$\begin{array}{c} g^{\beta_{i}}=g_{\vec{n}}^{\beta_{i}^{{}^{\prime }}}g_{\vec{n}}^{\theta_{i}}, \end{array}$$

$$\begin{array}{c} sk_{u}=\left(sk_{u1},sk_{u2}\right)=\left(Z_{u}^{r}/\prod_{j\in S}Z_{2^{n}-j+u},V^{\beta_{i}}\right). \end{array}$$

for the user *i* with identity *u* in the challenge set *S*. Finally ℬ sends all private keys *sk*_*u*_(*u* ∉ *S*) to A.

**Challenge**. A requests for the challenge and ℬ computes *Hdr* = (*U*, *U* ^*r*^). ℬ randomly chooses *b* ∈ {0, 1}. If *b* = 0, ℬ computes *K* = *W* ^*t*^, else *b* = 1, ℬ randomly chooses a key $K\in G_{2\vec{n}}$. Also, ℬ randomly chooses *b*_*u*_ for the user *i* with identity *u* ∈ *S*. And if *b*_*u*_ = 0, ℬ computes $K_{u}=e_{2}{\left(U,g_{\vec{n}}\right)}^{\beta_{i}\gamma }\cdot e_{2}{\left(U,V\right)}^{\theta_{i}}$, else *b*_*u*_ = 1, ℬ randomly chooses a personalized key $K_{u}\in G_{2\vec{n}}$. Finally ℬ gives A the challenge response (*Hdr*, *K*, {*K*_*u*_}_*u* ∈ *S*_). Apparently, the response (*Hdr*, *K*, {*K*_*u*_}_*u* ∈ *S*_) is valid. So ℬ simulates the real BEPM scheme for A perfectly.

**Guess**. A guesses *b*′ for *b* and ${\left\{b_{u}^{\prime }\right\}}_{u\in S}$ for {*b*_*u*_}_*u* ∈ *S*_. When ${\left\{b_{u}^{\prime }\right\}}_{u\in S}={\left\{b_{u}\right\}}_{u\in S}$ and *b*′ = *b*, it means the adversary A wins the game. $A_{win}$ indicates the event that the adversary A can guess the right value for *b* and {*b*_*u*_}_*u* ∈ *S*_. ${ℬ}_{win}$ indicates the event that the algorithm ℬ can solve the decisional *n*-HDHE problem. |*S*| is the total number of elements in set *S*. Hence, if *K* and {*K*_*u*_}_*u* ∈ *S*_ are right values, the probability of the event ${ℬ}_{win}$ occurring is:

$$\begin{array}{c} Pr\left[B_{win}\right]=Pr\left[B_{win}|A_{win}\right]\cdot Pr\left[A_{win}\right]+Pr\left[B_{win}|{\bar{A}}_{win}\right]\cdot Pr\left[{\bar{A}}_{win}\right] \end{array}$$

$$\begin{array}{c} =1\times \left(1/2\times 1/2^{\left|S\right|}+\epsilon \right)+1/2\times \left(1-\left(1/2\times 1/2^{\left|S\right|}+\epsilon \right)\right) \end{array}$$

$$\begin{array}{c} =1/4\times 1/2^{\left|S\right|}+1/2+\epsilon /2 \end{array}$$

Also, if *K* and {*K*_*u*_}_*u* ∈ *S*_ are random values chosen from $G_{2\vec{n}}$, which means the adversary A does not have the advantege *ϵ* to guess the *b* and {*b*_*u*_}_*u* ∈ *S*_, so the probability of the event ${ℬ}_{win}$ occurring is:

$$\begin{array}{c} Pr^{{}^{\prime }}\left[B_{win}\right]=Pr\left[B_{win}|A_{win}\right]\cdot Pr\left[A_{win}\right]+Pr\left[B_{win}|{\bar{A}}_{win}\right]\cdot Pr\left[{\bar{A}}_{win}\right] \end{array}$$

$$\begin{array}{c} =1\times 1/2\times 1/2^{\left|S\right|}+1/2\times \left(1-1/2\times 1/2^{\left|S\right|}\right) \end{array}$$

$$\begin{array}{c} =1/4\times 1/2^{\left|S\right|}+1/2 \end{array}$$

Above all, the advantage of ℬ to solve the decisional *n*-HDHE problem is: $Pr[{ℬ}_{win}]-Pr^{\prime }[{ℬ}_{win}]=\epsilon /2$.

However, the decisional *n*-HDHE assumption is a hard problem, so the advantage *ϵ* of ℬ is negligible. Hence, the advantage of the adversary A to break the BEPM scheme is negligible.

### Collusion resistant

In our security analysis, the adversary can get any private key of user *i* with identity *u* ∉ *S* while it can not get the right plaintext of Hdr*. It means any number of colluders can not get the right messages because they do not have any right private key.

### Comparison

In this section, we compare our scheme with Ohtake’s scheme [15] and the basic extension of Boneh’s scheme [6]. In Table 1, we use an integer *n* to represent the number of users in BEPM scheme. We claim that it is inefficient to send personalized messages in [6] while the header is changed to $\left(g_{\vec{n}}^{t},{\left(V\cdot \prod_{u\in S}Z_{2^{n}-u}\right)}^{t},{\left\{g_{\vec{n}}^{\beta_{u}}\right\}}_{u\in S}\right)$ and the ciphertext size is the number of |*S*| + 2 elements in group $G_{2\vec{n}}$ (|*S*| is the total number of elements in set *S*). Ohtake’s scheme extends the BGW [7] scheme by increasing the public key size from the number of 2*n* + 1 elements in group $G_{1}$ to 3*n* + 2 ($G_{1}$ is a group of prime order *p*). By comparing with Ohtake’s scheme, our scheme is identity-based and has a shorter public key size which is the number of log*n* elements of group $G_{\vec{n}}$, and our scheme removes the element *V*_2_ which is used in Ohtake’s scheme to encrypt personalized messages. And our scheme uses multilinear maps and keeps the ciphertext overhead and each user’s private key short. Hence, our scheme is more efficient than these two schemes.

**Table 1:** Comparison with the other schemes.

Scheme	**Mathematical theory**	**Public key size**	**Ciphertext length**
*Ohtake*	bilinear maps	3*n* + 2	2
*BWZ*14	multilinear maps	log*n*	|*S*| + 2
*Ourscheme*	multilinear maps	log*n*	2

Note: Here, we use the number of elements of groups to represent the public key size and ciphertext length.

## Application

Our BEPM scheme can be used to support personalized services in broadcast encryption while it has the following functions: first, our scheme can send a broadcast message by using the key *K*. Next, our scheme can send personalized messages by using the personalized key *K*_*u*_ to each user *u* ∈ *S*. In addition, the key management in our scheme is with low cost.

**Fig 1:**
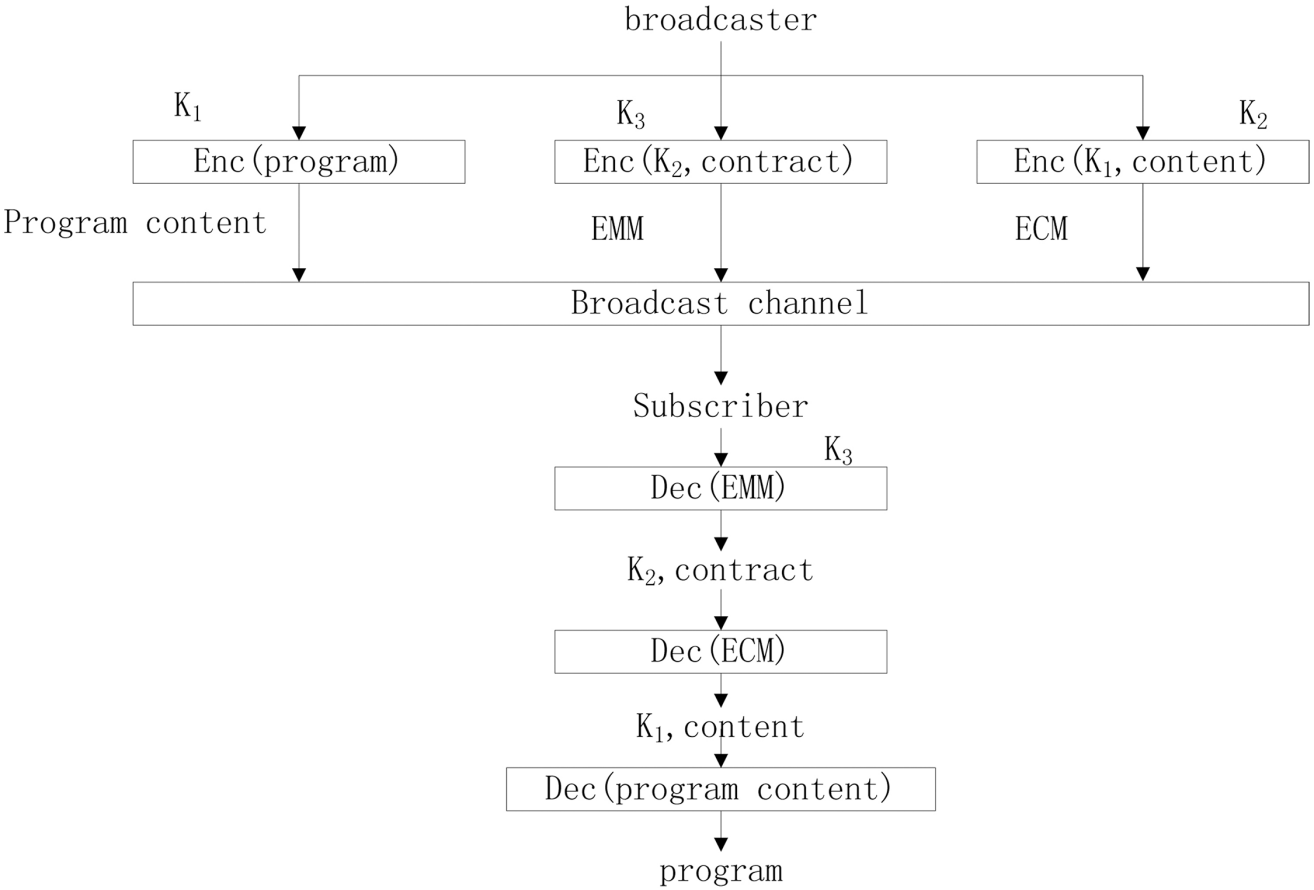
The work procedures of CAS.

As an important component of Digital TV Broadcasting (DVB), the CAS is a necessary and central condition for actualizing the service of pay-TV. The CAS can determine whether a digital receiver can transmit the specific broadcast programs to the users’ terminal with ensuring that only the paying users can get the selected TV programs. It is a necessary part of the digital television business, and it is also essential to the development of the digital television business. The Fig 1 shows the work procedures of CAS. The service provides ECM and EMM with stream of the same programs from different CAS to multiplex transmission channel. The decoder receives the detected ECM and EMM as the CAS requires. ECM is authorized control information, and it is a special form of electronic key signal and addressing channel information, and it is encrypted by sending end and then transmitted together with the signal. In receiving end, ECM is used to control the descrambler. EMM is authorized management information, which is information for an authorized user to descramble a business, and it is also encrypted by sending end and then transmitted together with the signal. In receiving end, EMM is used to open or close a single decoder or a group of descramblers. A broadcaster uses a scramble key *K*_1_ to encrypt content such as date, Media Access Control (MAC) address and program types. Then the broadcaster uses another key *K*_2_ to encrypt the scramble key and content information as ECM and transmits to all users. Finally, the broadcaster uses key *K*_3_ which is individual from other users to encrypt the key *K*_2_ and some contract information such as expire date as EMM and sends it to all users. Hence, we can apparently know that the content can only be descrambled by the user who has *K*_3_, which means the user is a valid subscriber to the program.

**Fig 2:**
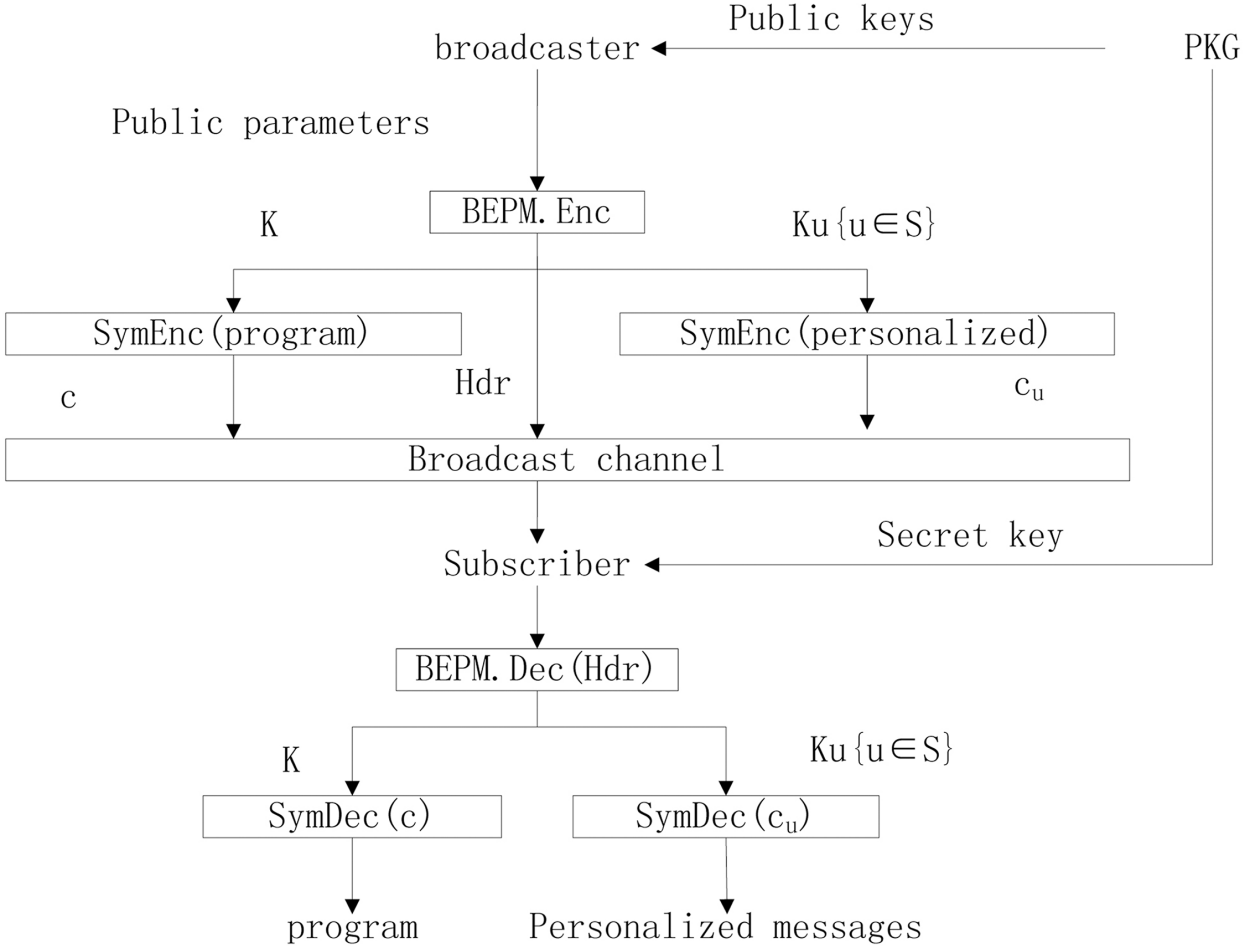
Our BEPM scheme used in CAS.

So the CAS is useful to transmit a broadcast message and personalized messages to each user of our BEPM scheme. But the broadcaster must manage all users’ key *K*_3_ while our scheme do not request the broadcaster to manage all users’ private keys. We can apply our BEPM scheme to CAS as Fig 2 shows. A broadcaster first computes the header, broadcast key *K* and personalized key *K*_*u*_ for any user *u* ∈ *S*. Then it uses *K* to encrypt a broadcast program content as a ciphertext *c* and broadcasts it. And it also uses *K*_*u*_ to encrypt the personalized message of any user *u* ∈ *S* as *c*_*u*_ and broadcasts it. A valid subscriber *u* ∈ *S* receives *c* and *c*_*u*_, it respectively uses *K* and *K*_*u*_ to decrypt *c* and *c*_*u*_ to get program content and personalized message as contract information.

## Results

In this paper, we construct an efficient BEPM scheme by using multilinear maps. Our scheme has the following advantages: first, the public key size in our scheme is shorter than any other existed schemes and the length of the ciphertext in our scheme is constant as well as all users’ private keys. Second, comparing with other general BE schemes, the broadcaster can not only send broadcast messages to all recipients but also send a personalized message to any specified user. Third, our BEPM scheme is statically secure and collusion resistant against any number of colluders. Last, it is efficient to apply our scheme to CAS which is the core of the popular pay-TV.

## References

[1] Amos Fiat, Moni Naor: Broadcast Encryption. CRYPT 1993. LNCS, vol. 773, pp.480–491. Springer, Heidelberg (1994)

[2] Xiubin Zou, Jinhai Xiang: Dynamic broadcast encryption scheme with revoking user. Wuhan University Journal of Natural Sciences, vol. 18, pp. 499–503. Springer, Heidelberg (2013) doi: 10.1007/s11859-013-0963-3

[3] Benny Chor, Amos Fiat, Moni Naor: Tracing traitors. In: 14th Annual International Cryptology Conference on Advances in Cryptology, pp. 257–270. Springer-Verlag, London (1994)

[4] Adi Shamir: Identity-Based Cryptosystems and Signature Schemes.Crypt 1985. LNCS, vol. 196, pp. 47–53. Springer, Heidelberg (1985)

[5] Aggelos Kiayias, Serdar Pehlivanoglu.: Encryption for Digital Content. Information Security. LNCS, vol. 52, pp. 35–105. Springer, Heidelberg (2010)

[6] Dan Boneh, Brent Waters, Mark Zhandry: Low Overhead Broadcast Encryption from Multilinear Maps. CRYPTO 2014. LNCS, vol. 8616, pp. 206–233. Springer, Heidelberg (2014)

[7] Dan Boneh, Craig Gentry, Brent Waters: Collusion Resistant Broadcast Encryption with Short Ciphertexts and Private Keys. CRYPTO 2005. LNCS, vol. 3621, pp. 258–275. Springer, Heidelberg (2005)

[8] Cécile Delerablée: Identity-Based Broadcast Encryption with Constant Size Ciphertexts and Private Keys. ASIACRYPT 2007. LNCS, vol. 4833, pp. 200–215. Springer, Heidelberg (2007)

[9] Gentry C., Waters B.: Adaptive security in broadcast encryption systems(with short ciphertexts). EUROCRYPT 2009. LNCS, vol. 5479, pp. 71–90. American Mathematical Society (2003)

[10] Yanli Ren, Shuozhong Wang, Xinpeng Zhang: Non-interactive Dynamic Identity-Based Broadcast Encryption without Random Oracles.Information and Communications Security. LNCS, vol. 7618, pp. 479–487. Springer, Heidelberg (2012)

[11] Boneh D., Silverberg A.: Applications of Multilinear Forms to Cryptography. Contemporary Mathematics, vol. 324, pp. 171–188. Springer, Heidelberg (2009)

[12] Dan Boneh, Mark Zhandry: Multiparty Key Exchange, Efficient Traitor Tracing, and More from Indistinguishability Obfuscation. CRYPTO 2014. LNCS, vol. 8616, pp. 480–499. Springer, Heidelberg (2014)

[13] Kaoru Kurosawa: Multi-recipient Public-Key Encryption with Shortened Ciphertext. Public Key Cryptography. LNCS, vol. 2274, pp. 48–63. Springer, Heidelberg (2002)

[14] Harunaga Hiwatari, Keisuke Tanaka, Tomoyuki Asano, Koichi Sakumoto: Multi-recipient Public-Key Encryption from Simulators in Security Proofs. Information Security and Privacy. LNCS, vol. 5594, pp. 293–308. Springer, Heidelberg (2009)

[15] Go Ohtake, Goichiro Hanaoka, Kazuto Ogawa: Efficient Broadcast Encryption with Personalized Messages. Provable Security. LNCS, vol. 6402, pp. 214 –228. Springer, Heidelberg (2010)

